# Trends in treatment patterns and survival outcomes in advanced non-small cell lung cancer: a Canadian population-based real-world analysis

**DOI:** 10.1186/s12885-022-09342-5

**Published:** 2022-03-10

**Authors:** Robert Carroll, Margherita Bortolini, Alan Calleja, Robin Munro, Shiying Kong, Melinda J. Daumont, John R. Penrod, Khalid Lakhdari, Laure Lacoin, Winson Y. Cheung

**Affiliations:** 1 Centre for Observational Research & Data Sciences, Bristol Myers Squibb, Uxbridge, UK; 2 Real World Solutions, IQVIA, London, UK; 3 Department of Oncology, University of Calgary, Calgary, AB Canada; 4 Worldwide Health Economics & Outcomes Research, Bristol Myers Squibb, Braine-l’Alleud, Belgium; 5 Worldwide Health Economics & Outcomes Research, Bristol Myers Squibb, Princeton, NJ USA; 6 Health Economics and Market Access Oncology, Bristol Myers Squibb, Saint-Laurent, QC Canada; 7 Epi-Fit, Bordeaux, Nouvelle-Aquitaine, France; 8 Department of Medical Oncology, Tom Baker Cancer Centre, University of Calgary, Calgary, AB Canada

**Keywords:** Immune checkpoint inhibitors, Immunotherapy, Non-small cell lung cancer, Population based, Real-world evidence, Retrospective cohort study, Survival, Treatment patterns

## Abstract

**Background:**

As part of the multi-country I-O Optimise research initiative, this population-based study evaluated real-world treatment patterns and overall survival (OS) in patients treated for advanced non-small cell lung cancer (NSCLC) before and after public reimbursement of immuno-oncology (I-O) therapies in Alberta province, Canada.

**Methods:**

This study used data from the Oncology Outcomes (O2) database, which holds information for ~ 4.5 million residents of Alberta. Eligible patients were adults newly diagnosed with NSCLC between January 2010 and December 2017 and receiving first-line therapy for advanced NSCLC (stage IIIB or IV) either in January 2010-March 2016 (pre–I-O period) or April 2016-June 2019 (post–I-O period). Time periods were based on the first public reimbursement of I-O therapy in Alberta (April 2017), with a built-in 1-year lag time before this date to allow progression to second-line therapy, for which the I-O therapy was indicated. Kaplan–Meier methods were used to estimate OS.

**Results:**

Of 2244 analyzed patients, 1501 (66.9%) and 743 (33.1%) received first-line treatment in the pre–I-O and post–I-O periods, respectively. Between the pre–I-O and post–I-O periods, proportions of patients receiving chemotherapy decreased, with parallel increases in proportions receiving I-O therapies in both the first-line (from < 0.5% to 17%) and second-line (from 8% to 47%) settings. Increased use of I-O therapies in the post–I-O period was observed in subgroups with non-squamous (first line, 15%; second line, 39%) and squamous (first line, 25%; second line, 65%) histology. First-line use of tyrosine kinase inhibitors also increased among patients with non-squamous histology (from 26% to 30%). In parallel with these evolving treatment patterns, median OS increased from 10.2 to 12.1 months for all patients (*P* < 0.001), from 11.8 to 13.7 months for patients with non-squamous histology (*P* = 0.022) and from 7.8 to 9.4 months for patients with squamous histology (*P* = 0.215).

**Conclusions:**

Following public reimbursement, there was a rapid and profound adoption of I-O therapies for advanced NSCLC in Alberta, Canada. In addition, OS outcomes were significantly improved for patients treated in the post–I-O versus pre–I-O periods. These data lend support to the emerging body of evidence for the potential real-world benefits of I-O therapies for treatment of patients with advanced NSCLC.

**Supplementary Information:**

The online version contains supplementary material available at 10.1186/s12885-022-09342-5.

## Background

Lung cancer is the leading cause of cancer death both worldwide and in Canada, where it was estimated to be responsible for 25% of all cancer deaths in 2020 [1, 2]. Moreover, current estimates suggest that 1 in 18 men and 1 in 20 women in Canada will die of lung cancer [2]. Non-small cell lung cancer (NSCLC) is the most commonly diagnosed lung cancer type worldwide, representing 80–90% of all diagnoses, and predominantly presents as lung adenocarcinoma [3].

While platinum-based chemotherapy regimens have long been the standard treatment option for patients with advanced NSCLC, the past 2 decades have witnessed a profound transformation of the treatment paradigm for patients with lung cancer. In the early-to-mid 2000s, the first approvals of epidermal growth factor receptor (EGFR) tyrosine kinase inhibitors (TKIs) combined with a better understanding of the role of “driver” mutations in the pathogenesis and progression of NSCLC marked the start of the targeted therapy era [4, 5]. Subsequently, TKIs targeting other driver mutations, such as anaplastic lymphoma kinase (*ALK*), *ROS1*, *BRAF V600E, *or* NTRK1/2/3* alterations have been approved for patients with NSCLC tumors harboring these mutations [6–10]. More recently, immuno-oncology (I-O) therapies, primarily immune checkpoint inhibitors targeting the programmed death-1 (PD-1)/programmed death ligand 1 (PD-L1) pathway, have demonstrated improved survival outcomes versus standard chemotherapy in randomized clinical trials and have emerged as recommended first- and second-line treatments in Europe and North America for patients with advanced NSCLC without actionable driver mutations [3, 11–15].

Given the step change in the availability of new treatments for advanced NSCLC, population-level analyses investigating temporal trends in treatment patterns and outcomes could shed light on whether changes in clinical practice are translating into improved patient survival. However, such analyses rely on robust and detailed real-world data on cancer patient treatment and outcomes. Data of such depth are scarce and typically rely on linkage between databases focused on population-level disease monitoring (e.g., cancer registries) and detailed clinical data from electronic medical records (EMR) or claims sources.

I-O Optimise is a multi-country, observational research initiative that utilizes real-world databases to provide valuable insights on the evolving treatment landscape for thoracic malignancies [16]. The Oncology Outcomes (O2) database, which collects data on a variety of malignancies from the population of Alberta province in Canada, is one such database. The aim of the current study was to evaluate real-world treatment patterns and survival outcomes for patients treated for advanced NSCLC between 2010 and 2019 in Alberta using the O2 database. The analyses were also specifically designed to explore trends in patterns of treatment and patient survival before and after public reimbursement of I-O therapies for the treatment of advanced NSCLC.

## Methods

### Database overview

The O2 database, in partnership with Alberta Health Services and Cancer Care Alberta, holds information for the entire province of Alberta, representing a population of approximately 4.5 million residents. The database comprises a set of data from the Alberta Cancer Registry, also maintained by Alberta Health Services, linked to other relevant datasets (e.g., Alberta Vital Statistics). O2 is a multisource database that integrates registry, EMR, administrative, claims and pharmacy data from 17 cancer centers, including two tertiary centers, four regional centers and 11 community sites. The Alberta Cancer Registry is responsible for recording and maintaining data on all new primary cancers, as well as all cancer deaths occurring within Alberta. Since 2004, the registry has employed the International Classification of Diseases for Oncology, 3rd edition (ICD-O-3) to classify all cancers by site and morphology and the International Classification of Diseases and Related Health Problems, 10th Revision (ICD-10) to record cancer deaths and cancer-related health problems. Of note, information was not available in the O2 database on patient performance status, mutational status (e.g., the presence or absence of *EGFR* or *ALK* mutations), or PD-L1 expression level.

### Study and analysis populations

In this population-based study, patients were eligible if they had a new diagnosis of lung cancer (ICD-10 codes C33 [malignant neoplasm of the trachea] or C34 [malignant neoplasm of bronchus and lung]) between 1 January 2010 and 31 December 2017 and were at least 18 years of age at diagnosis. Patients were excluded if they had an ICD-O-3 morphology code indicating small cell lung cancer or a concomitant primary tumor within 5 years before and 1.5 years after their lung cancer diagnosis.

The current analysis is focused on patients meeting the study inclusion/exclusion criteria who received a first-line therapy for advanced NSCLC (tumor, nodes, metastasis [TNM] stage IIIB or IV) either between 1 January 2010 and 31 March 2016 (referred to as the pre–I-O period) or between 1 April 2016 and 30 June 2019 (referred to as the post–I-O period). These time periods were based on the date of first public reimbursement approval for an I-O therapy in Alberta (the April 2017 approval of nivolumab for patients with advanced or metastatic NSCLC who progressed on or after first-line cytotoxic chemotherapy [17]), with a built-in lag time of 1 year before this date to allow patients to progress to second-line therapy, for which the I-O therapy was indicated. The post–I-O period also captures subsequent I-O therapy approvals in Alberta (the February 2018 approval of pembrolizumab for patients with locally advanced or previously untreated metastatic NSCLC and for patients with metastatic NSCLC whose tumors express PD-L1 and who progressed on or after cytotoxic chemotherapy [18, 19]), as well as off-label, clinical trial, and early-access use of various I-O therapies.

In addition to patients initially diagnosed with stage IIIB or IV NSCLC, the analysis population also included “progressed patients,” defined as those patients initially diagnosed at earlier stages of the disease (TNM stages I, II, or IIIA) but who subsequently received systemic anticancer therapy within the aforementioned time periods (see Additional file 1 for relevant eligibility criteria).

### Statistical methodology

Descriptive statistics were used for reporting of patient demographic and clinical characteristics and treatment patterns. The Charlson Comorbidity Index, a score characterizing patient comorbidity burden [20, 21], was derived based on the prevalence of comorbidities prior to index treatment. Systemic anticancer therapies received by patients were coded in the O2 database and identified for this analysis using the World Health Organization (WHO) Anatomical Therapeutic Chemical (ATC) classification system [22]. Overall survival (OS) was estimated using the Kaplan–Meier method and was defined as the time from the start of first-line therapy for advanced NSCLC (for “progressed patients,” this was the date of receipt of systemic anticancer therapy per Additional file 1) to the date of death from any cause during the study period. Patients who remained alive through the study were censored at the date of loss to follow-up or at the end of the study period (30 June 2019), whichever occurred first. Differences in OS between the pre–I-O and post–I-O periods were assessed using the log-rank test. Statistical analysis was performed using SAS 9.4 (SAS Institute, Inc., Cary, NC).

## Results

### Patients

Overall, 2244 patients met the study inclusion/exclusion criteria and received a first-line therapy for advanced NSCLC between 1 January 2010 and 30 June 2019. Across the entire study period, most patients were initially diagnosed with advanced disease (stage IIIB, 12.0%; stage IV, 75.2%) and had non-squamous (NSQ) histology (70.4%). The most common sites of distant metastases at diagnosis were non-liver/non-adrenal visceral (25.9%) and bone (16.8%). The majority of patients had a Charlson Comorbidity Index of 0–1 (54.1%), and the most frequent non–cancer-related comorbidities were chronic pulmonary disease (35.5%) and diabetes with or without related complications (16.2%).

Of the overall cohort, 1501 (66.9%) and 743 (33.1%) patients received first-line treatment in the pre–I-O and post–I-O periods, respectively. Patient and disease characteristics were generally well balanced between the time periods for all patients and for subpopulations with NSQ or squamous (SQ) histology (Table 1). However, there was a trend toward an increase in the proportion of patients initially diagnosed at stages I–IIIA (from 10.6% in the pre–I-O period to 17.4% in post–I-O period), concomitant with a trend toward a decrease in the proportion diagnosed at stage IV (from 77.0% to 71.5%). There was also a trend toward an increase in the proportions of patients with NSQ (from 68.4% to 74.4%) and SQ (from 15.0% to 17.4%) histology, concomitant with a trend toward a decrease in the proportion with NSCLC “not otherwise specified” histology (from 15.1% to 7.0%).

**Table 1:** Characteristics of patients receiving a first-line treatment for advanced NSCLC in the pre– and post–I-O periods

	**Pre**–**I-O** **(1 January 2010–31 March 2016)**			**Post**–**I-O** **(1 April 2016–30 June 2019)**		
	**All (*N* = 1501)**^**a**^	**NSQ (*n* = 1026)**	**SQ (*n* = 225)**	**All (*N* = 743)**^**b**^	**NSQ (*n* = 553)**	**SQ (*n* = 129)**
**Age,**^**c**^** years**						
Median Q1–Q3 Range	65 58–72 26–91	64 56–72 26–91	68 61–72 39–85	68 60–74 27–96	66 59–74 27–96	69 63–73 43–86
**Female, ***n*** (%)**	763 (50.8)	555 (54.1)	85 (37.8)	388 (52.2)	314 (56.8)	42 (32.6)
**Initial TNM stage, ***n*** (%)**						
I–IIIA^d^	159 (10.6)	89 (8.7)	46 (20.4)	129 (17.4)	85 (15.4)	38 (29.5)
IIIB	186 (12.4)	102 (9.9)	53 (23.6)	83 (11.2)	46 (8.3)	31 (24.0)
IV	1156 (77.0)	835 (81.4)	126 (56.0)	531 (71.5)	422 (76.3)	60 (46.5)
**Location of metastases, ***n*** (%)**						
Visceral, excl. adrenal/liver	390 (26.0)	297 (28.9)	35 (15.6)	192 (25.8)	157 (28.4)	22 (17.1)
Bone	259 (17.3)	194 (18.9)	26 (11.6)	118 (15.9)	100 (18.1)	12 (9.3)
Liver	144 (9.6)	102 (9.9)	15 (6.7)	43 (5.8)	35 (6.3)	5 (3.9)
Brain	132 (8.8)	109 (10.6)	< 5	88 (11.8)	75 (13.6)	7 (5.4)
Lymph	114 (7.6)	76 (7.4)	17 (7.6)	58 (7.8)	43 (7.8)	9 (7.0)
Adrenal	80 (5.3)	61 (5.9)	8 (3.6)	40 (5.4)	34 (6.1)	< 5
**CCI, ***n*** (%)**						
0	477 (31.8)	331 (32.3)	69 (30.7)	229 (30.8)	178 (32.2)	34 (26.4)
1	345 (23.0)	222 (21.6)	66 (29.3)	164 (22.1)	114 (20.6)	39 (30.2)
2	159 (10.6)	102 (9.9)	32 (14.2)	76 (10.2)	54 (9.8)	17 (13.2)
3	202 (13.5)	147 (14.3)	23 (10.2)	93 (12.5)	69 (12.5)	17 (13.2)
4 +	318 (21.2)	224 (21.8)	35 (15.6)	181 (24.4)	138 (25.0)	22 (17.1)

Unless otherwise indicated, all characteristics were recorded at time of diagnosis. Data were masked when patient numbers for an individual category were greater than zero but less than 5

Abbreviations: *CCI* Charlson Comorbidity Index; *I-O* immuno-oncology; *NOS* not otherwise specified; *NSCLC* non-small cell lung cancer; *NSQ* non-squamous; *Q* quartile; *SQ* squamous; *TNM* tumor, nodes, metastasis

^a^Patients had NSQ (*n* = 1026), SQ (*n* = 225), NOS (*n* = 226) or Other (*n* = 24) histology

^b^Patients had NSQ (*n* = 553), SQ (*n* = 129), NOS (*n* = 52) or Other (*n* = 9) histology

^c^Age recorded at start of first-line treatment for advanced NSCLC

^d^Patients with TNM stage I–IIIA NSCLC at diagnosis represent “progressed patients” who subsequently received systemic anticancer therapy during the study period (see Additional file 1)

### Treatment patterns

#### First-line treatment

**Fig. 1:**
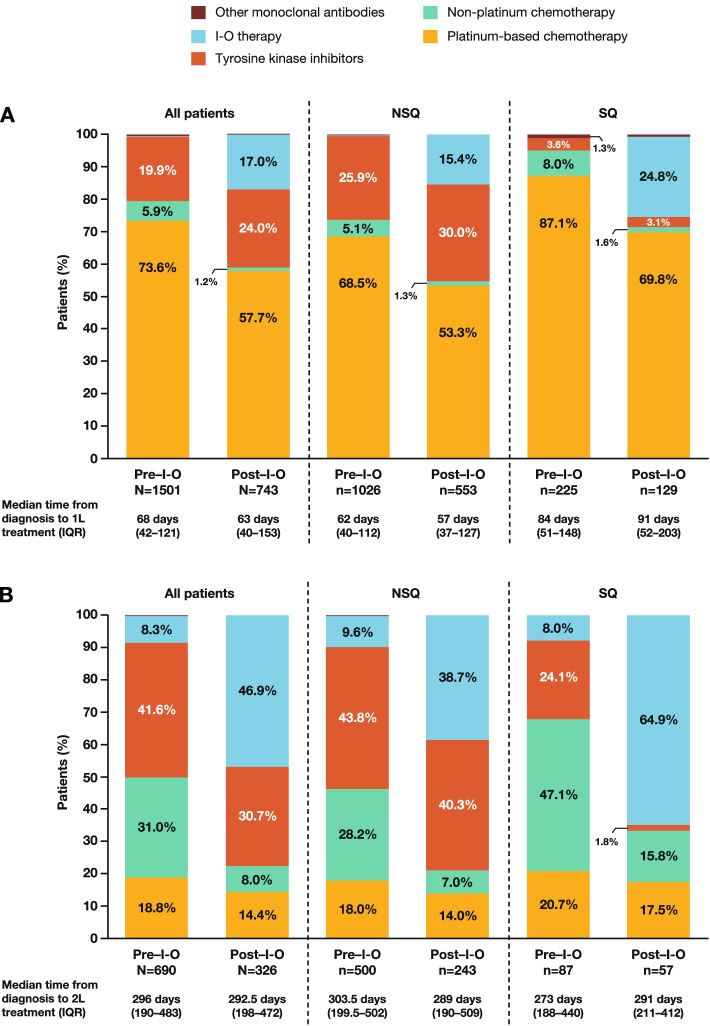
First-line **A** and second-line **B** treatments received by patients with advanced NSCLC in the pre– and post–I-O periods. Percentages for treatment categories are only displayed if > 1%. Abbreviations: *1L* first line; *2L* second line; *I-O* immuno-oncology; *IQR* interquartile range; *NSCLC* non-small cell lung cancer; *NSQ* non-squamous; *SQ* squamous

First-line treatment classes administered during the pre– and post–I-O periods for all patients and for patients with NSQ or SQ histology are shown in Fig. 1A. Regardless of the population of interest, the most common first-line treatments in both time periods were platinum-based chemotherapies. In the pre–I-O period, the most common platinum-based regimens were carboplatin plus vinorelbine among patients with NSQ (13.5%) and carboplatin plus gemcitabine among patients with SQ (24.9%); in the post–I-O period, they were carboplatin plus pemetrexed among patients with NSQ (27.1%) and carboplatin plus gemcitabine among patients with SQ (27.1%). However, the overall proportion of patients receiving platinum-based chemotherapy regimens decreased from 73.6% in the pre–I-O period to 57.7% in the post–I-O period. Similarly, although only administered as first-line treatment to a relatively small proportion of patients, the use of non-platinum chemotherapy also decreased, from 5.9% in the pre–I-O period to 1.2% in the post–I-O period.

In parallel with the decreased use of first-line chemotherapy, the proportion of patients receiving I-O therapies increased (Fig. 1A). As expected, in the pre–I-O period first-line use of these therapies was negligible, but during the post–I-O period they were administered as first-line treatment to 17.0% of all patients, 15.4% of patients with NSQ histology, and 24.8% of patients with SQ histology. The first-line I-O therapies administered in the post–I-O period were primarily anti–PD-1/PD-L1 monotherapies including atezolizumab, durvalumab, nivolumab, and pembrolizumab, with most patients (86.5%) receiving nivolumab or pembrolizumab monotherapy. A small proportion of those receiving first-line I-O therapy (5.6%) were treated with durvalumab in combination with the anti-CTLA-4 inhibitor tremelimumab, either as the duotherapy alone or further combined with chemotherapies. Use of TKIs also increased between the pre– and post–I-O periods (from 19.9% to 24.0%) with the greatest increase in patients with NSQ histology (Fig. 1A); the most common TKI administered was gefitinib in both time periods.

Median time from diagnosis to first-line treatment was longer for patients with SQ NSCLC than those with NSQ histology. However, time from diagnosis to first-line treatment did not differ substantially between the pre– and post–I-O periods, regardless of the population of interest (Fig. 1A).

#### Second-line treatment

Overall, 1016 patients received a second-line treatment during the study period (Table 2): 690 in the pre–I-O period and 326 in the post–I-O period. Characteristics of the patients receiving a second-line treatment were relatively consistent with those for patients receiving first-line treatment and were also similar between the pre– and post–I-O periods. Furthermore, patterns of first-line treatment for these patients mirrored the overall changes in first-line treatments described above (Table 2).

**Table 2:** Characteristics of patients receiving a second-line treatment for advanced NSCLC in the pre– and post–I-O periods

	**Pre**–**I-O** **(1 January 2010–31 March 2016)**			**Post**–**I-O** **(1 April 2016–30 June 2019)**		
	**All (*N* = 690)**^**a**^	**NSQ (*n* = 500)**	**SQ (*n* = 87)**	**All (*N* = 326)**^**b**^	**NSQ (*n* = 243)**	**SQ (*n* = 57)**
**Age,**^**c**^** years**						
Median Q1–Q3 Range	65 57–72 29–91	65 56.5–72.5 29–91	66 62–72 39–80	66 59–73 31–90	65 58–73 31–90	70 64–72 43–84
**Female, ***n*** (%)**	380 (55.1)	296 (59.2)	36 (41.4)	177 (54.3)	148 (60.9)	16 (28.1)
**Initial TNM stage, ***n*** (%)**						
I–IIIA^d^	66 (9.6)	36 (7.2)	17 (19.5)	38 (11.7)	22 (9.1)	15 (26.3)
IIIB	86 (12.5)	59 (11.8)	19 (21.8)	38 (11.7)	21 (8.6)	14 (24.6)
IV	538 (78.0)	405 (81.0)	51 (58.6)	250 (76.7)	200 (82.3)	28 (49.1)
**Location of metastases, ***n*** (%)**						
Visceral, excl. adrenal/liver	164 (23.8)	132 (26.4)	12 (13.8)	77 (23.6)	63 (25.9)	9 (15.8)
Bone	94 (13.6)	80 (16.0)	6 (6.9)	50 (15.3)	43 (17.7)	< 5
Liver	47 (6.8)	35 (7.0)	< 5	16 (4.9)	13 (5.3)	< 5
Brain	44 (6.4)	39 (7.8)	< 5	39 (12.0)	34 (14.0)	< 5
Lymph	39 (5.7)	26 (5.2)	7 (8.0)	21 (6.4)	15 (6.2)	< 5
Adrenal	25 (3.6)	17 (3.4)	< 5	13 (4.0)	13 (5.3)	0 (0.0)
**CCI, ***n*** (%)**						
0	234 (33.9)	171 (34.2)	25 (28.7)	108 (33.1)	89 (36.6)	14 (24.6)
1	165 (23.9)	118 (23.6)	30 (34.5)	72 (22.1)	50 (20.6)	19 (33.3)
2	69 (10.0)	47 (9.4)	14 (16.1)	27 (8.3)	18 (7.4)	6 (10.5)
3	91 (13.2)	68 (13.6)	8 (9.2)	39 (12.0)	26 (10.7)	11 (19.3)
4 +	131 (19.0)	96 (19.2)	10 (11.5)	80 (24.5)	60 (24.7)	7 (12.3)
**First-line treatment, ***n*** (%)**						
Platinum-based chemotherapy	491 (71.2)	331 (66.2)	77 (88.5)	189 (58.0)	123 (50.6)	46 (80.7)
Tyrosine kinase inhibitor	154 (22.3)	141 (28.2)	< 5	104 (31.9)	100 (41.2)	< 5
Non-platinum chemotherapy	38 (5.5)	23 (4.6)	7 (8.0)	5 (1.5)	< 5	< 5
I-O therapy	< 5	< 5	0 (0.0)	27 (8.3)	16 (6.6)	7 (12.3)
Other monoclonal antibodies	6 (0.9)	< 5	< 5	< 5	0 (0.0)	< 5
**Duration of first-line treatment, ***n*** (%)**						
< 1 month	136 (19.7)	78 (15.6)	27 (31.0)	41 (12.6)	20 (8.2)	15 (26.3)
1– < 3 months	328 (47.5)	228 (45.6)	54 (62.1)	132 (40.5)	90 (37.0)	34 (59.6)
3– < 6 months	103 (14.9)	84 (16.8)	< 5	71 (21.8)	60 (24.7)	6 (10.5)
6– < 12 months	61 (8.8)	53 (10.6)	< 5	49 (15.0)	44 (18.1)	< 5
≥ 12 months	62 (9.0)	57 (11.4)	< 5	32 (9.8)	28 (11.5)	< 5
Unknown	0 (0.0)	0 (0.0)	0 (0.0)	< 5	< 5	0 (0.0)

Unless otherwise indicated, all characteristics were recorded at time of diagnosis. Data were masked when patient numbers for an individual category were greater than zero but less than 5

Abbreviations: *CCI* Charlson Comorbidity Index; *I-O* immuno-oncology; *NOS* not otherwise specified; *NSCLC* non-small cell lung cancer; *NSQ* non-squamous; *Q* quartile; *SQ* squamous; *TNM* tumor, nodes, metastasis

^a^Patients had NSQ (*n* = 500), SQ (*n* = 87), NOS (*n* = 93) or Other (*n* = 10) histology

^b^Patients had NSQ (*n* = 243), SQ (*n* = 57), NOS (*n* = 25) or Other (*n* =  < 5) histology

^c^Age recorded at start of second-line treatment for advanced NSCLC

^d^Patients with TNM stage I–IIIA NSCLC at diagnosis represent “progressed patients” who subsequently received systemic anticancer therapy during the study period (see Additional file 1)

The second-line treatment classes administered in the pre– and post–I-O periods are shown in Fig. 1B. In the pre–I-O period, TKIs (most commonly erlotinib, regardless of histology) and non-platinum chemotherapies (most commonly single-agent pemetrexed for patients with NSQ or single-agent docetaxel for patients with SQ) were the most frequently administered second-line therapies, accounting for more than 70% of treated patients. During the post–I-O period, use of second-line TKIs and non-platinum chemotherapy decreased, with the reduction for non-platinum chemotherapy most notable (a decline from 31.0% to 8.0% of all treated patients). In parallel with these declines, the use of second-line I-O therapy increased substantially from 8.3% of all treated patients during the pre–I-O period to 46.9% during the post–I-O period; the increase was most notable in patients with SQ histology (an increase from 8.0% to 64.9% of all treated patients) (Fig. 1B). Most patients receiving second-line I-O therapy in the post–I-O period (94.1%) received nivolumab or pembrolizumab monotherapy. Again, a small proportion of the patients administered second-line I-O therapies (3.3%) received I-O–based combination regimens.

Median time from diagnosis to second-line treatment did not differ substantially between patients diagnosed with NSQ or SQ NSCLC, nor between the pre– and post–I-O periods, regardless of the population of interest (Fig. 1B).

#### Treatment sequencing

**Fig. 2:**
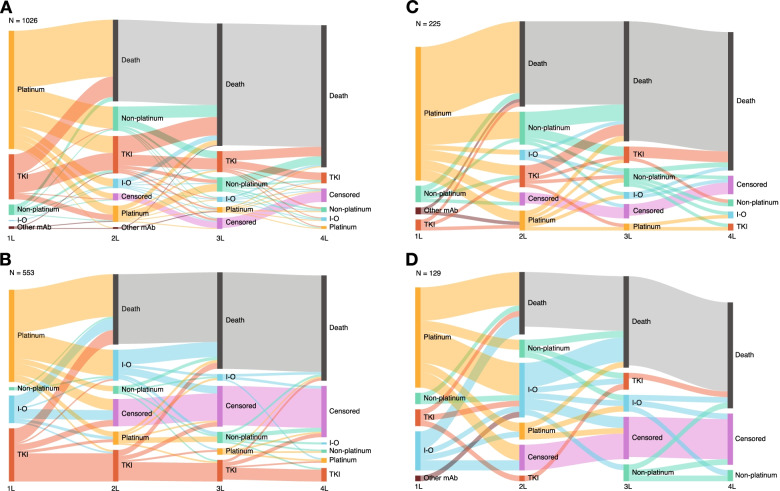
Treatment sequencing for patients who received first-line treatment for advanced NSCLC with NSQ **A** and **B** or SQ **C** and **D** histology in the pre– and post–I-O periods, respectively. Data for categories reflecting treatment of between 1 and 5 patients are masked and have been allocated a standardized line thickness in each of the Sankey diagrams, relative to the respective total number of patients. As such, the thickness of lines may not always correspond as patients transition through the sequence of treatments. Abbreviations: *1L* first line; *2L* second line; *3L* third line; *4L* fourth line; *I-O* immuno-oncology; *mAb* monoclonal antibody; *Non-platinum* non-platinum chemotherapy; *NSCLC* non-small cell lung cancer; *NSQ* non-squamous; *Platinum* platinum-based chemotherapy; *SQ* squamous; *TKI* tyrosine kinase inhibitor

Treatment sequencing from the first to the fourth line of therapy during the pre– and post–I-O periods is shown for patients with NSQ and SQ histology in Fig. 2. Regardless of time period or histology, a substantial proportion of treated patients died during or after first-line treatment (pre–I-O period range, 47.6–54.2%; post–I-O period range, 38.0–40.3%). Likewise, in both time periods, only a relatively small number of patients went on to receive third- or fourth-line treatment, irrespective of histology. Assessment of differences between the time periods in the proportions dying during or after first-line therapy and the proportions receiving third- or fourth-line therapy is confounded by the fact that the maximum possible observation interval was shorter for the post–I-O period (~ 3 years) than the pre–I-O period (~ 6 years), which resulted in a greater proportion of censored patients in the post–I-O period. For example, during the pre–I-O period, between 3.7% and 7.1% of patients were censored prior to administration of second-line treatment; during the post–I-O period, between 15.7% and 17.8% were censored.

### Overall survival

**Fig. 3:**
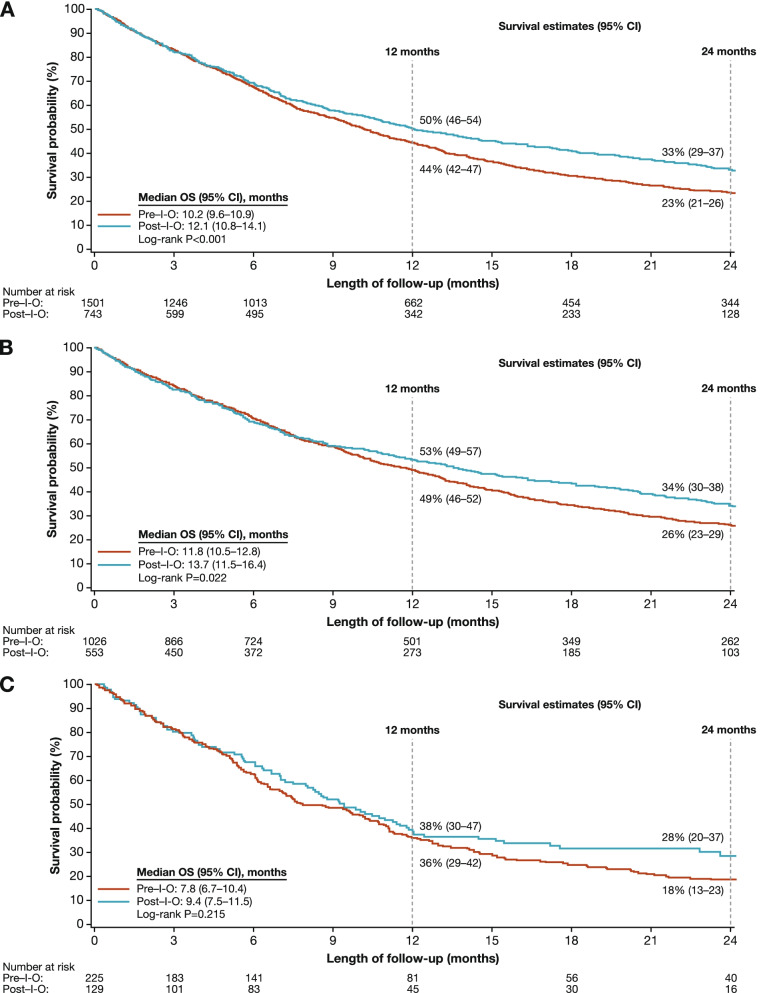
Overall survival in patients who received first-line treatment for advanced NSCLC in the pre– and post–I-O periods: **A** all patients, **B** patients with NSQ histology, and **C** patients with SQ histology. Abbreviations: *CI* confidence interval; *I-O* immuno-oncology; *NSCLC* non-small cell lung cancer; *NSQ* non-squamous; *OS* overall survival; *SQ* squamous.

Median OS (95% confidence interval [CI]) from start of first-line therapy was 10.2 months (9.6–10.9) for patients treated in the pre–I-O period and 12.1 months (10.8–14.1) for patients treated in the post–I-O period, with 2-year OS estimates of 23% and 33%, respectively (Fig. 3A); the difference between OS in the two time periods was statistically significant (*P* < 0.001). Among patients with NSQ histology, median OS (95% CI) was 11.8 months (10.5–12.8) for the pre–I-O period and 13.7 months (11.5–16.4) for the post–I-O period, with 2-year OS estimates of 26% and 34%, respectively (Fig. 3B); again, the difference between OS in the two periods was statistically significant (*P* = 0.022). In patients with SQ histology, median OS (95% CI) was 7.8 months (6.7–10.4) for the pre–I-O period and 9.4 months (7.5–11.5) for the post–I-O period, with 2-year OS rates of 18% and 28%, respectively (Fig. 3C); in this population, the difference between OS in the two periods was not statistically significant (*P* = 0.215).

## Discussion

Using data from the Canadian O2 database, the current population-based analysis shows a rapid adoption of I-O therapies for the treatment of advanced NSCLC in Alberta, Canada after public reimbursement of these agents. In the post–I-O period, there was a profound increase in the proportion of patients receiving an I-O therapy in either the first-line (17% vs < 0.5% in the pre–I-O period) or second-line (47% vs 8% in the pre–I-O period) setting. In addition, OS outcomes were significantly improved for patients treated in the post–I-O period, with a third of patients estimated to be alive 2 years after the start of first-line treatment compared with less than a quarter of those treated during the pre–I-O period.

The observed uptake of I-O therapies in Alberta emulates real-world data on the adoption of these agents elsewhere. For example, in a large-scale analysis of patients treated at US academic and community oncology practices, more than 60% of eligible patients had received anti–PD-1 therapy within 4 months of US Food and Drug Administration (FDA) approval of the agent [23]. Likewise, two separate analyses of data from US community oncology clinics both showed a striking increase in the proportion of patients receiving first- or second-line anti–PD-1/PD-L1 therapy for NSCLC after FDA approval [24, 25]. Finally, in a previous US-based analysis of real-world treatment patterns and outcomes before and after FDA approval of anti–PD-1/PD-L1 therapies for NSCLC, approximately half (48.8%) of patients receiving a second-line therapy after FDA approval received an anti–PD-1/PD-L1 therapy [26]; this proportion is noticeably similar to that observed in the current study for second-line I-O therapy (46.9%).

The OS improvements seen in the post–I-O period are encouraging. Although establishing a direct link between the observed uptake of I-O therapy and the improved survival is beyond the scope of the current study, it could be hypothesized that increased use of I-O therapies contributed at least in part. Indeed, evidence from randomized clinical trials would suggest that the evolution of treatment patterns observed in this study (i.e., a shift from using mostly chemotherapy-based regimens to an increased proportion receiving immune checkpoint inhibitors) would have a positive influence on survival outcomes in both the first- and second-line settings [12–15, 27–29]. Moreover, in the aforementioned US-based analysis of real-world treatment patterns and outcomes before and after anti–PD-1/PD-L1 therapy approval, the substantial post-approval increase in second-line use of anti–PD-1/PD-L1 agents was also accompanied by significant improvements in OS [26]. In addition, another recent publication using data from Surveillance, Epidemiology, and End Results (SEER) areas in the US showed evidence of a consistent decrease in NSCLC mortality between 2013 and 2016 that exceeded the decline in incidence of this subtype, with the authors suggesting that this resulted from the increased use of newly available targeted (i.e., TKIs) and I-O therapies over that time period [30].

As acknowledged above, the current analysis was not explicitly designed to determine the influence of increased I-O therapy use on OS, and several factors should be considered when interpreting the observed outcomes. First, other treatment pattern changes were noted over the study period. For example, in addition to I-O therapy, TKIs were also more frequently used as first-line therapy in the post–I-O versus pre–I-O period, with proportions receiving first-line TKI increasing from 20% to 24% overall and from 26% to 30% among patients with NSQ NSCLC. This usage pattern likely reflects updates to international guidelines to expand routine testing for various driver mutations, and, as a consequence, increased testing and detection rates [31]. Although the overall proportion of patients receiving second-line TKIs decreased between the pre– and post–I-O period (from 42% to 31%), the likely contribution of increased first-line TKI use on the observed improvement in OS cannot be discounted. Similarly, it is possible that other advancements occurring over the period of the study, for example in patient management and palliative care, could have also contributed to the survival improvements seen in the post–I-O period.

Second, it is important to acknowledge that this was not a pure “no I-O versus I-O” comparison. Approximately 8% of patients who received first-line treatment in the pre–I-O period went on to receive an I-O therapy as second-line therapy. Moreover, as can be seen in the Sankey diagrams (Fig. 2), a small number of patients who received a first-line therapy in the pre–I-O period subsequently received an I-O therapy in the third- or fourth-line setting. This second- or later-line I-O therapy use would likely have increased survival of patients in the pre–I-O cohort, potentially leading to overestimated OS outcomes for that period.

Finally, the maximum possible observation interval differed between the time periods (~ 6 years for the pre–I-O period vs ~ 3 years for the post–I-O period), which may have influenced the observed outcomes. Indeed, the shorter observation interval for the post–I-O period resulted in a greater proportion of censored patients. As these censored patients were most likely to be those patients still alive at the end of the study, this could mean the survival outcomes in the post–I-O period were underestimated. Additional analyses with longer post–I-O follow-up will help further evaluate differences between the time periods in OS outcomes.

In the current analysis, treatment patterns and survival outcomes differed between those with NSQ and SQ histology. One of the most noteworthy differences was that TKI use was almost exclusively restricted to patients with NSQ NSCLC in the post–I-O period. In parallel, there was a greater adoption of I-O therapy among patients with SQ versus NSQ NSCLC during this time period. These histological differences are somewhat expected based on the fact that (i) international clinical practice guidelines recommend I-O therapies, but not TKIs, for patients not carrying driver mutations [3, 11]; (ii) the same practice guidelines either do not recommend, or only weakly recommend, molecular testing for SQ NSCLC [3, 11]; and (iii) the literature suggests that TKI-associated driver mutations are very rare in Caucasian patients with SQ histology [32–35], although marginally higher in Asian patients [35, 36].

Survival outcomes also differed by tumor histology with patients with NSQ NSCLC having longer OS than those with SQ NSCLC, regardless of the time period. Better survival outcomes in patients with NSQ versus SQ histology have been observed in numerous randomized clinical trials and other real-world studies [12, 13, 15, 26, 37, 38]. Importantly, however, the improvements in median OS between the pre– and post–I-O periods observed in the current analysis were numerically similar between patients with NSQ or SQ NSCLC. Moreover, although the difference in OS between the pre– and post–I-O periods was not statistically significant for patients with SQ histology, this is likely a result of the relatively small patient population.

In addition to some of the above considerations, the findings of the current analysis should be interpreted in the context of additional strengths and limitations. For example, the findings are strengthened by the large overall patient population; the long duration of analysis, which captures nearly a decade of treatment patterns and survival outcomes data; the inclusion of both broad (registry) and detailed (EMR, claims, pharmacy) real-world data; and the inclusion of data from tertiary and regional centers, as well as community sites. Limitations of the study include the fact that certain patient or tumor characteristics that could have influenced outcomes, such as performance status, smoking status, patient ethnicity, mutational status, and PD-L1 expression, were unavailable in this data source or are not routinely collected in Canada. It is also important to acknowledge that while the available baseline characteristics of patients between the two periods appeared balanced, the observed differences between the two periods in OS could be driven both by observed as well as unobserved confounders.

## Conclusions

The current analysis, representing some of the first Canadian data on population-level treatment patterns and I-O therapy trends, showed a rapid and profound real-world adoption of I-O therapies for the treatment of advanced NSCLC. Over the same study period, significant improvements in patient OS were also observed, although the analysis was not designed to determine the specific driver(s) of these improvements. Despite these encouraging developments, many treated patients with advanced NSCLC still die during or after first-line therapy, highlighting a continuing need for improved therapeutic approaches. Recent promising results combining I-O therapies with angiogenesis inhibitors, other I-O agents, and/or chemotherapies, as well as ongoing investigations of other novel combinatorial treatment approaches [28, 39–44], will hopefully continue to expand the armamentarium against advanced NSCLC and further improve patient survival.

## Supplementary Information

Below is the link to the electronic supplementary material.

**Additional file1:** Inclusion of “progressed patients” in analysis population

## Data Availability

The data from this study are not publicly available and no data sharing is planned. Patient level data cannot to be shared due to regulatory and confidentiality reasons. Aggregate results from the study are presented in this manuscript. Further questions on data sharing should be directed to the corresponding author (Dr Winson Y. Cheung).

## Abbreviations

1L: First line
2L: Second line
ALK: Anaplastic lymphoma kinase
ATC: Anatomical Therapeutic Chemical
CCI: Charlson Comorbidity Index
CI: Confidence interval
EGFR: Epidermal growth factor receptor
EMR: Electronic medical records
FDA: US Food and Drug Administration
ICD-10: International Classification of Diseases and Related Health Problems, 10th Revision
ICD-O-3: International Classification of Diseases for Oncology, 3rd edition
I-O: Immuno-oncology
IQR: Interquartile range
mAb: Monoclonal antibody
Non-platinum: Non-platinum chemotherapy
NOS: Not otherwise specified
NSCLC: Non-small cell lung cancer
NSQ: Non-squamous
O2: Oncology Outcomes
OS: Overall survival
PD-1: Programmed death-1
PD-L1: Programmed death ligand 1
Platinum: Platinum-based chemotherapy
Q: Quartile
SEER: Surveillance, Epidemiology, and End Results
SQ: Squamous
TKI: Tyrosine kinase inhibitor
TNM: Tumor, nodes, metastasis
WHO: World Health Organization

## References

[1] Sung H, Ferlay J, Siegel RL, Laversanne M, Soerjomataram I, Jemal A (2021). Global cancer statistics 2020: GLOBOCAN estimates of incidence and mortality worldwide for 36 cancers in 185 countries. CA Cancer J Clin.

[2] Canadian Cancer Society. Lung cancer statistics. https://www.cancer.ca/en/cancer-information/cancer-type/lung/statistics/?region=pe. Accessed 7 Mar 2022.

[3] Planchard D, Popat S, Kerr K, Novello S, Smit EF, Faivre-Finn C (2018). Metastatic non-small cell lung cancer: ESMO Clinical Practice Guidelines for diagnosis, treatment and follow-up.. Ann Oncol.

[4] Herbst RS, Bunn PA (2003). Targeting the epidermal growth factor receptor in non-small cell lung cancer. Clin Cancer Res.

[5] Pao W, Miller VA (2005). Epidermal growth factor receptor mutations, small-molecule kinase inhibitors, and non-small-cell lung cancer: current knowledge and future directions. J Clin Oncol.

[6] Frampton JE (2013). Crizotinib: a review of its use in the treatment of anaplastic lymphoma kinase-positive, advanced non-small cell lung cancer. Drugs.

[7] Kazandjian D, Blumenthal GM, Chen HY, He K, Patel M, Justice R (2014). FDA approval summary: crizotinib for the treatment of metastatic non-small cell lung cancer with anaplastic lymphoma kinase rearrangements. Oncologist.

[8] Lin JJ, Shaw AT (2017). Recent advances in targeting ROS1 in lung cancer. J Thorac Oncol.

[9] Weart TC, Miller KD, Simone CB (2018). Spotlight on dabrafenib/trametinib in the treatment of non-small-cell lung cancer: place in therapy. Cancer Manag Res.

[10] Frampton JE (2021). Entrectinib: a review in NTRK+ solid tumours and ROS1+ NSCLC. Drugs..

[11] National Comprehensive Cancer Network (NCCN). Clinical Practice Guidelines in Oncology: non-small cell lung cancer - version 2.2021. https://www.nccn.org/guidelines/guidelines-detail?category=1&id=1450. Accessed 15 Mar 2021.

[12] Borghaei H, Paz-Ares L, Horn L, Spigel DR, Steins M, Ready NE (2015). Nivolumab versus docetaxel in advanced nonsquamous non-small-cell lung cancer. N Engl J Med.

[13] Brahmer J, Reckamp KL, Baas P, Crino L, Eberhardt WE, Poddubskaya E (2015). Nivolumab versus docetaxel in advanced squamous-cell non-small-cell lung cancer. N Engl J Med.

[14] Reck M, Rodriguez-Abreu D, Robinson AG, Hui R, Csoszi T, Fulop A (2016). Pembrolizumab versus chemotherapy for PD-L1-positive non-small-cell lung cancer. N Engl J Med.

[15] Rittmeyer A, Barlesi F, Waterkamp D, Park K, Ciardiello F, von Pawel J (2017). Atezolizumab versus docetaxel in patients with previously treated non-small-cell lung cancer (OAK): a phase 3, open-label, multicentre randomised controlled trial. Lancet.

[16] Ekman S, Griesinger F, Baas P, Chao D, Chouaid C, O'Donnell JC (2019). I-O Optimise: a novel multinational real-world research platform in thoracic malignancies. Future Oncol.

[17] pCODR. Provincial funding summary: nivolumab (Opdivo) for metastatic non-small cell lung cancer (pCODR 10069). https://www.cadth.ca/sites/default/files/pcodr/pcodr_provfund_nivolumab_opdivo_nsclc.pdf. Accessed 25 Mar 2021.

[18] pCODR. Provincial funding summary: pembrolizumab (Keytruda) for advanced non-small cell lung carcinoma (first line) (pCODR 10101). https://www.cadth.ca/sites/default/files/pcodr/PembrolizumabKeytruda_AdvancedNSCLCfirstline10101_ProvFunding.pdf. Accessed 25 Mar 2021.

[19] pCODR. Provincial funding summary: pembrolizumab (Keytruda) for non-small cell lung cancer (second line or beyond) (pCODR 10077). https://www.cadth.ca/sites/default/files/pcodr/pcodr_provfund_pembrolizumab_keytruda_nsclc_2lnbeyond.pdf. Accessed 25 Mar 2021.

[20] Charlson ME, Pompei P, Ales KL, MacKenzie CR (1987). A new method of classifying prognostic comorbidity in longitudinal studies: development and validation. J Chronic Dis.

[21] Quan H, Sundararajan V, Halfon P, Fong A, Burnand B, Luthi JC (2005). Coding algorithms for defining comorbidities in ICD-9-CM and ICD-10 administrative data. Med Care.

[22] WHO Collaborating Centre for Drug Statistics Methodology. ATC/DDD Index 2021. https://www.whocc.no/atc_ddd_index/. Accessed 30 November 2021.

[23] O'Connor JM, Fessele KL, Steiner J, Seidl-Rathkopf K, Carson KR, Nussbaum NC (2018). Speed of adoption of immune checkpoint inhibitors of programmed cell death 1 protein and comparison of patient ages in clinical practice vs pivotal clinical trials. JAMA Oncol..

[24] Khozin S, Abernethy AP, Nussbaum NC, Zhi J, Curtis MD, Tucker M (2018). Characteristics of real-world metastatic non-small cell lung cancer patients treated with nivolumab and pembrolizumab during the year following approval. Oncologist.

[25] Nadler E, Arondekar B, Aguilar KM, Zhou J, Chang J, Zhang X (2021). Treatment patterns and clinical outcomes in patients with advanced non-small cell lung cancer initiating first-line treatment in the US community oncology setting: a real-world retrospective observational study. J Cancer Res Clin Oncol.

[26] Schwartzberg L, Korytowsky B, Penrod JR, Zhang Y, Le TK, Batenchuk C (2019). Real-world clinical impact of immune checkpoint inhibitors in patients with advanced/metastatic non-small cell lung cancer after platinum chemotherapy. Clin Lung Cancer..

[27] Mok TSK, Wu YL, Kudaba I, Kowalski DM, Cho BC, Turna HZ (2019). Pembrolizumab versus chemotherapy for previously untreated, PD-L1-expressing, locally advanced or metastatic non-small-cell lung cancer (KEYNOTE-042): a randomised, open-label, controlled, phase 3 trial. Lancet.

[28] Hellmann MD, Paz-Ares L, Bernabe Caro R, Zurawski B, Kim SW, Carcereny Costa E (2019). Nivolumab plus ipilimumab in advanced non-small-cell lung cancer. N Engl J Med.

[29] Borghaei H, Gettinger S, Vokes EE, Chow LQM, Burgio MA, de Castro Carpeno J (2021). Five-year outcomes from the randomized, phase III trials CheckMate 017 and 057: nivolumab versus docetaxel in previously treated non-small-cell lung cancer. J Clin Oncol.

[30] Howlader N, Forjaz G, Mooradian MJ, Meza R, Kong CY, Cronin KA (2020). The effect of advances in lung-cancer treatment on population mortality. N Engl J Med.

[31] Pennell NA, Arcila ME, Gandara DR, West H (2019). Biomarker testing for patients with advanced non-small cell lung cancer: real-world issues and tough choices. Am Soc Clin Oncol Educ Book.

[32] Chatziandreou I, Tsioli P, Sakellariou S, Mourkioti I, Giannopoulou I, Levidou G (2015). Comprehensive molecular analysis of NSCLC; clinicopathological associations. PLoS One..

[33] Skov BG, Hogdall E, Clementsen P, Krasnik M, Larsen KR, Sorensen JB (2015). The prevalence of EGFR mutations in non-small cell lung cancer in an unselected Caucasian population. APMIS.

[34] Zhao W, Choi YL, Song JY, Zhu Y, Xu Q, Zhang F (2016). ALK, ROS1 and RET rearrangements in lung squamous cell carcinoma are very rare. Lung Cancer.

[35] Han B, Tjulandin S, Hagiwara K, Normanno N, Wulandari L, Laktionov K (2017). EGFR mutation prevalence in Asia-Pacific and Russian patients with advanced NSCLC of adenocarcinoma and non-adenocarcinoma histology: the IGNITE study. Lung Cancer.

[36] Zhang Q, Zhu L, Zhang J (2015). Epidermal growth factor receptor gene mutation status in pure squamous-cell lung cancer in Chinese patients. BMC Cancer.

[37] Scagliotti G, Hanna N, Fossella F, Sugarman K, Blatter J, Peterson P (2009). The differential efficacy of pemetrexed according to NSCLC histology: a review of two Phase III studies. Oncologist.

[38] Soares M, Antunes L, Redondo P, Borges M, Hermans R, Patel D (2020). Real-world treatment patterns and survival outcomes for advanced non-small cell lung cancer in the pre-immunotherapy era in Portugal: a retrospective analysis from the I-O Optimise initiative. BMC Pulm Med.

[39] Paz-Ares L, Luft A, Vicente D, Tafreshi A, Gumus M, Mazieres J (2018). Pembrolizumab plus chemotherapy for squamous non-small-cell lung cancer. N Engl J Med.

[40] Socinski MA, Jotte RM, Cappuzzo F, Orlandi F, Stroyakovskiy D, Nogami N (2018). Atezolizumab for first-line treatment of metastatic nonsquamous NSCLC. N Engl J Med.

[41] Ready N, Hellmann MD, Awad MM, Otterson GA, Gutierrez M, Gainor JF (2019). First-line nivolumab plus ipilimumab in advanced non-small-cell lung cancer (CheckMate 568): outcomes by programmed death ligand 1 and tumor mutational burden as biomarkers. J Clin Oncol.

[42] Arrieta O, Barron F, Ramirez-Tirado LA, Zatarain-Barron ZL, Cardona AF, Diaz-Garcia D (2020). Efficacy and safety of pembrolizumab plus docetaxel vs docetaxel alone in patients with previously treated advanced non-small cell lung cancer: the PROLUNG phase 2 randomized clinical trial. JAMA Oncol.

[43] Paz-Ares L, Ciuleanu TE, Cobo M, Schenker M, Zurawski B, Menezes J (2021). First-line nivolumab plus ipilimumab combined with two cycles of chemotherapy in patients with non-small-cell lung cancer (CheckMate 9LA): an international, randomised, open-label, phase 3 trial. Lancet Oncol.

[44] Passiglia F, Reale ML, Cetoretta V, Novello S (2021). Immune-checkpoint inhibitors combinations in metastatic NSCLC: new options on the horizon?. Immunotargets Ther.

