# Supplementary material for: Trends in treatment patterns and survival outcomes in advanced non-small cell lung cancer: a Canadian population-based real-world analysis
Source: BMC Cancer. 2022 Mar 10;22:255. doi: 10.1186/s12885-022-09342-5 (PMC8908553; doi:10.1186/s12885-022-09342-5)
Supplement: Supplementary file 1 — Additional file1: Inclusion of “progressed patients” in analysis population [file 12885_2022_9342_MOESM1_ESM.pdf]

Additional File 1.

Inclusion of "progressed patients" in analysis population

The current analyses included "progressed patients," defined as those meeting the general study inclusion/exclusion criteria as well as the following additional eligibility criteria:

- Diagnosed with tumor, nodes, metastasis (TNM) stage I, II, or IIIA non-small cell lung cancer (NSCLC) and received systemic anticancer therapy (SACT) alone as initial treatment
    - If the initial treatment was SACT alone, then that was captured as the patient's first-line treatment and any subsequent therapy was captured as second-line treatment
- Diagnosed with TNM stage I, II, or IIIA NSCLC and received one of the following therapies as initial treatment and then received a subsequent SACT regimen:
    - Surgery + adjuvant SACT
    - Surgery + adjuvant radiotherapy and SACT
    - Neoadjuvant SACT + surgery
    - Neoadjuvant radiotherapy and SACT + surgery
    - Radiotherapy followed by SACT
    - SACT followed by radiotherapy
    - Chemoradiotherapy

  For these patients, the subsequent SACT regimen was captured as the patient's first-line treatment and any subsequent therapy as second-line treatment

Of note, treatment with SACT as described above was used as a proxy for progression to advanced disease (stage IIIB or IV) in "progressed patients"; no confirmatory restaging data were available.
